# Activation of KLF1 Enhances the Differentiation and Maturation of Red Blood Cells from Human Pluripotent Stem Cells

**DOI:** 10.1002/stem.2562

**Published:** 2017-01-19

**Authors:** Cheng‐Tao Yang, Rui Ma, Richard A. Axton, Melany Jackson, A. Helen Taylor, Antonella Fidanza, Lamin Marenah, Jan Frayne, Joanne C. Mountford, Lesley M. Forrester

**Affiliations:** ^1^ Centre for Regenerative MedicineUniversity of EdinburghEdinburghUnited Kingdom; ^2^ Institute of Cardiovascular & Medical Sciences, University of GlasgowGlasgowUnited Kingdom; ^3^ Scottish National Blood Transfusion ServiceScotlandUnited Kingdom; ^4^ Department of BiochemistryUniversity of BristolUnited Kingdom

**Keywords:** Erythroid differentiation, Induced pluripotent stem cells, Transcription factors, Gene delivery systems in vivo or in vitro

## Abstract

Blood transfusion is widely used in the clinic but the source of red blood cells (RBCs) is dependent on donors, procedures are susceptible to transfusion‐transmitted infections and complications can arise from immunological incompatibility. Clinically‐compatible and scalable protocols that allow the production of RBCs from human embryonic stem cells (hESCs) and induced pluripotent stem cells (iPSCs) have been described but progress to translation has been hampered by poor maturation and fragility of the resultant cells. Genetic programming using transcription factors has been used to drive lineage determination and differentiation so we used this approach to assess whether exogenous expression of the Erythroid Krüppel‐like factor 1 (EKLF/KLF1) could augment the differentiation and stability of iPSC‐derived RBCs. To activate KLF1 at defined time points during later stages of the differentiation process and to avoid transgene silencing that is commonly observed in differentiating pluripotent stem cells, we targeted a tamoxifen‐inducible KLF1‐ER^T2^ expression cassette into the *AAVS1* locus. Activation of KLF1 at day 10 of the differentiation process when hematopoietic progenitor cells were present, enhanced erythroid commitment and differentiation. Continued culture resulted the appearance of more enucleated cells when KLF1 was activated which is possibly due to their more robust morphology. Globin profiling indicated that these conditions produced embryonic‐like erythroid cells. This study demonstrates the successful use of an inducible genetic programing strategy that could be applied to the production of many other cell lineages from human induced pluripotent stem cells with the integration of programming factors into the *AAVS1* locus providing a safer and more reproducible route to the clinic. Stem Cells *2017;35:886–897*

**Significance Statement**

Production of red blood cells from human pluripotent stem cells in the laboratory could solve many of the problems associated with blood transfusion but clinical trials have been hampered by the poor maturation status and fragility of differentiated cells. Here, we demonstrate the successful use of an inducible transcription factor programing strategy that results in the enhanced differentiation and maturation of red blood cells. This strategy could be applied to the production of many other cell lineages from pluripotent stem cells with the integration of programming factors into a safer harbor locus providing a safer and more reproducible route to the clinic.

## Introduction

The generation of an unlimited supply of red blood cells (RBCs) from human pluripotent stem cells (hPSCs) such as human embryonic stem cells (hESCs) or induced pluripotent stem cells (iPSCs), could alleviate many of the current problems facing the blood transfusion services such as transfusion transmitted infection, donor supply and immune compatibility. Scalable, clinically compatible protocols to produce erythroid cells from hPSCs have been developed but progress to translation has been hampered by the lack of terminal maturation of the resultant cell. In contrast to RBCs generated in vitro from adult bone marrow or mobilised peripheral blood CD34^+^ progenitors cells, erythroid cells produced from both hESCs and iPSCs have a fragile morphology, a poor enucleation rates and express embryonic and foetal rather than adult globin 1, 2, 3, 4, 5, 6, 7.

Transcription factors are arguably the most important route to controlling cell type identity as they drive lineage‐specific genes associated with their functional properties 8. Transcription factor programming has been used to direct hESC/iPSC differentiation into distinct cell types such as cardiomyocytes and neurons 9, 10. Enhanced expression of transcription factors known to be involved in the development and maintenance of the hematopoietic system such as SCL/TAL1, RUNX1, HOXA9, or HOXB4 have been used to increase the production of hematopoietic stem/progenitor cells from hESCs/iPSCs 11, 12, 13, 14, 15, 16 and four transcription factors (GATA1, LMO2, SCL/TAL1, and cMYC) directly converted fibroblast into primitive erythroid progenitors 17.

Erythroid Kruppel‐like factor 1 (EKLF/KLF1) is a zinc finger DNA binding protein that plays a critical role in regulating the expression of genes involved in erythroid cell identity and function including those involved in heme biosynthesis, red cell membrane stability and adult globin 18, 19. Coassociations of KLF1‐regulated genes at specialized nuclear hotspots is thought to optimize the coordinated transcriptional control 20

Detailed analyses of mouse mutants demonstrated that *Klf1* deficiency results in defects in hemoglobin metabolism and membrane stability and that KLF1‐null erythroid cells in the fetal liver have an abnormal morphology with many retaining their nuclei 21, 22, 23, 24, 25. Deficiencies in *KLF1* have also been associated with human disease 26, 27. For example, a missense mutation in *KLF1* results in a dominant‐negative congenital dyserythropoietic anemia 28. Reduced activity of *KLF1* has been associated with the rare blood group In (Lu) phenotype with amino acid substitutions within zinc finger domains predicted to abolish the interactions of KLF1 with downstream targets 29, 30, 31. Genomic sequencing has uncovered the fact that a broad range human red cell disorders are caused by variants in *KLF1* 32.

We noted that KLF1 was expressed at a lower level in erythroid cells derived from hESCs compared to adult CD34^+^‐derived cells and, given its importance in erythroid maturation, we hypothesized this low level of expression of *KLF1* might be one reason for their lack of maturity. We first assessed the effects of constitutive expression of KLF1 and noted a significant reduction in the proliferative capacity of differentiating hESCs and a high variability in expression and stability of the transgene. We, therefore, developed a strategy where we could induce activity of KLF1 at later time‐points during the differentiation process after hematopoietic progenitor cells (HPCs) had formed by generating and testing a human KLF1‐ER^T2^ fusion protein. To achieve a consistent and physiological level of expression and to avoid transgene silencing, we employed the “safe harbor” approach by integrating the inducible KLF1‐ER^T2^ transgene into the *AAVS1* locus 33, 34, 35.

We show for the first time that the inducible activation of KLF1 at a defined time point during the differentiation of both hESC and iPSCs enhanced erythroid commitment and differentiation. Continued culture of KLF1‐activated cells resulted in a more robust morphology and a higher proportion of detectable enucleated cells. Globin profiling indicated that erythroid cells produced under these conditions had an embryonic‐like phenotype.

## Materials and Methods

### Plasmid Construction

cDNAs encoding human wild type KLF1 or mutant R328L KLF1 31 were amplified by polymerase chain reaction (PCR) and cloned into the EcoRI‐digested pCAG‐IRES‐puro plasmid (pCAG‐SIP). Tamoxifen inducible KLF1‐ER^T2^ and R328L‐ER^T2^ fusion cassettes were generated by recombineering (Supporting Information Fig. S1B, S1D, S1E). CAG‐HA‐KLF1‐ER^T2^‐PolyA was cloned into the multiple cloning site of the pZDonor‐AAVS1 Puromycin vector (PZD0020, Sigma‐Aldrich, Gillingham, UK, http://www.sigmaaldrich.com/).

### Production of iPSCS from ORhesus Negative Individuals

Dermal fibroblasts were obtained from blood group O Rhesus negative individuals by R Biomedical Ltd, Edinburgh, UK, (http://www.rbiomedical.com) under REC 1/AL/0020 ethical approval. Fibroblasts were reprogrammed to iPSCs using an episomal strategy with the transcription factors, *OCT4, KLF2 SOX2,* and *cMYC* 8 (http://roslincells.com). Characterization of the SFCi55 cell line used in this study included flow cytometry for key pluripotency and differentiation markers (Supporting Information Fig. S2A, S2B). Chromosomal spreads revealed a normal 46XX karyotype that was then confirmed by SNP analysis (data not shown). Hematopoietic differentiation of SFCi55 compared favorably to H1 hESCs (data not shown) and other published iPSC lines (Supporting Information Fig. S2C).

### Maintenance and Differentiation of hESC and iPSCs

hESC and iPSCs were maintained in STEMPRO hESC SFM (Thermo Fisher Scientific Life Sciences, Waltham, MA, http://www.thermofisher.com) containing 20 ng/ml bFGF (FGF2) (R&D Systems, Abingdon, U.K., https://www.rndsystems.com) on CTS CELLstart Substrate (Thermo Fisher Scientific Life Sciences) and passaged (1:4) when 70%‐80% confluent using STEMPRO EZPassage (Thermo Fisher Scientific Life Sciences, Waltham, MA, http://www.thermofisher.com). Hematopoietic differentiation was carried out in a step‐wise, serum‐, and feeder‐free protocol as described in detail previously 15, 36

### Transfection of hESC and iPSCs

H1 hESC or iPSCs were fed with STEMPRO hESC SFM containing 20 ng/ml bFGF, and 10 μM Rock inhibitor (Y‐27632, Calbiochem, Darmstadt, Germany. http://www.merckmillipore.com) was added at least 1 hour prior to electroporation as described previously 11, 37. Single cell suspensions were generated using Accutase (Thermo Fisher Scientific Life Sciences), washed and resuspended (10^7^ cells per 0.5 ml) in Dulbecco's phosphate‐buffered saline without Ca^2+^ and Mg^2+^ (DPBS) and electroporated with 30 μg of linearized vector (BioRad Hemel Hempstead, UK http://www.bio-rad.com, Gene pulser; 320V 250 μF). Cells were plated on CTS CELLstart substrate in STEMPRO hESC SFM containing 20 ng/ml bFGF and 10 μM Rock inhibitor and 0.6 μg/ml puromycin for 10 days then resistant colonies were picked, expanded, and screened by PCR and Western blotting.

### K562 Cell Maintenance and Electroporation

K562 cells were seeded at 10^5^/ml in DMEM medium (Thermo Fisher Scientific Life Sciences) supplemented with 10% fetal calf serum, 2 mM sodium pyruvate (Thermo Fisher Scientific Life Sciences), 1% nonessential amino acids (Thermo Fisher Scientific Life Sciences), and 0.1 mM β‐mercaptoethanol (Thermo Fisher Scientific Life Sciences) and passaged every 2‐3 days. K562 cells (10^7^ cells in 700 μl DPBS) were electroporated (BioRad, Gene pulser; 320 V, 500 μF), then pools of cells were selected in 2.0 μg/ml puromycin (Sigma Aldrich) 2 days later. Hemin (50 μM) (Sigma Aldrich) was added to the cultures to induce differentiation then cells were harvested and analyzed after 4 days.

### COS7 Cell Maintenance and Transfection

COS7 cells were maintained in GMEM medium (Thermo Fisher Scientific Life Sciences) supplemented with 10% fetal calf serum, 2 mM sodium pyruvate (Thermo Fisher Scientific Life Sciences), 1% nonessential amino acids (Thermo Fisher Scientific Life Sciences), and 0.1 mM β‐mercaptoethanol (Thermo Fisher Scientific Life Sciences) and passaged at 1:5 ratio. Cells were seeded at 5 × 10^4^/well in a 6‐well‐plate and transfected with 2.5 μg of DNA plasmid using the Xfect Transfection Reagent (Clontech, Saint‐Germain‐en‐Laye, France. http://www.clontech.com).

### Quantitative Reverse‐Transcriptase Polymerase Chain Reaction

RNA was extracted using RNeasy Mini Kit (QIAGEN), and reverse transcription was performed by High‐Capacity cDNA Reverse Transcription Kit (Thermo Fisher Scientific Life Sciences) following the manufacturer's instructions. To normalize cDNA quantity, GAPDH was used as reference gene. PCR reactions were carried out in triplicate using Applied Biosystems 7500 Fast Real‐Time PCR System and data was analyzed on SDS v1.4 software (Thermo Fisher Scientific Life Sciences).

### Protein Extraction and Western Blotting

Cells were lysed in RIPA buffer (Thermo Fisher Scientific Life Sciences) for total protein extraction. For nuclear fractionation, the cell pellet was resuspended in 0.2 ml of Swelling Buffer (5 mM PIPES, pH 8.0; 85 mM KCL; 0.5% NP40; protease inhibitor cocktail) for 20 minutes on ice. After spinning at 1,500 rpm at 4°C, the cytoplasmic supernatant was removed. The nuclear pellet was resuspended in 0.3 ml of lysis buffer (20 mM Hepes, pH 7.6; 1.5 mM MgCl2; 350 mM KCl; 0.2 mM EDTA; 20% Gycerol; 0.25% NP40; 0.5 mM DTT; protease inhibitor cocktail; Benzonase) and gently shaken at 4°C for 1 hour. The nuclear fraction was collected after centrifuged at 13,000 rpm, 4°C, for 30 minutes and stored at −80°C. Proper amount of protein lysates were electrophoresed on 4%‐20% Ready Gel (BioRad), transferred to nitrocellulose membranes (10402580, Whatman, Sigma Aldrich) and probed with anti‐HA tag (631207; Clontech), anti‐KLF1 (sc14034, Santa Cruz, CA USA www.scbt.com), anti‐GAPDH (AF5718, R&D) antibodies or LaminB1 (ab16048, abcam, Cambridge, UK, http://www.abcam.com). Antibody binding was detected using the appropriate horseradish peroxidase‐conjugated IgG (HAF008, R&D Systems, Abingdon, U.K., https://www.rndsystems.com; sc‐2020, SantaCruz) visualized by the WesternSure ECL Substrate (LI‐COR, Cambridge, UK, https://www.licor.com).

### CFU‐C Assay

Day 10 differentiating cells (5 × 10^3^ or 10^4^) were plated into 1.5 ml of MethoCult (04435, Stem Cell Technologies, Cambridge, UK, https://www.stemcell.com) in 35 mm low attachment dishes (Greiner, Stonehouse, UK, https://www.gbo.com), incubated at 37°C in a humid chamber then scored for hematopoietic colony formation 12‐15 days later.

### Flow Cytometry

10^5^ differentiating cells were harvested in phosphate‐buffered saline (PBS) containing 1% bovine serum albumin (BSA) (PBS/BSA) and centrifuged at 200 g for 5 minutes. Cell pellets were resuspended and mixed with the appropriate volume of antibody, CD34‐PE (12‐0349‐41, eBioscience, eBioscience Ltd., Hatfield, UK, http://www.ebioscience.com/), CD43‐APC (17‐0439‐42, eBioscience), CD235a‐FITC (11‐9987‐80, eBioscience), and CD71‐APC (17‐0719‐42, eBioscience), to a final volume of 100 μl PBS/BSA, incubated for 30 minutes then analyzed on a LSR Fortessa (BD Biosciences, Oxford, UK, http://www.bdbiosciences.com/) using FACS Diva. The proportion of enucleated cells present in the culture was assessed using CD235a‐FITC, CD71‐APC antibodies, LIVE/DEAD Fixable Near‐IR Stain (L10119, Thermo Fisher Scientific) and Hoechst dye (NucBlue, Thermo Fisher Scientific). Live CD235a^+^ cells were first gated, then anti‐CD71 and Hoeschst were used to define erythroblasts (CD71^+^/Hoechst^+^), nucleated RBCs (CD71^−^/Hoechst^+^) and enucleated RBCs (CD71^−^/Hoechst^−^) (Supporting Information Fig. S7).

### Immunofluorescence Staining

COS7 cells were fixed in 4% formaldehyde/PBS for 15 minutes and permeabilized in 0.5% Triton‐X 100/PBS (PBST) and successively incubated for 1 hour with rabbit anti‐human KLF1 (sc14034, Santa Cruz), goat anti–rabbit IgG‐FITC (F0382‐1ML, SIGMA‐ALDRICH) antibodies, and DAPI (4',6‐Diamidino‐2‐phenylindole; SIGMA‐ALDRICH). Stained cells were analyzed using a Zeiss Observer microscope and processed with AxioVision and ImageJ software.

### Morphological Analysis

5 × 10^4^ erythroid cells were resuspended in 0.2 ml PBS, loaded in cytospin slide chamber, and centrifuged at 500 rpm for 10 minutes. Rapid Romanowksy staining of air‐dried slides was performed according to manufacturer's instructions (HS705, TCS biosciences, Buckinghamshire, UK, http://www.tcsbiosciences.co.uk).

### High‐Performance Liquid Chromatography

High‐performance liquid chromatography (HPLC) globin chain separation was performed using a protocol modified from Lapillonne et al. 38. Briefly, cells were washed three times in PBS, lysed in 50 μl water by three rapid freeze‐thaw cycles and centrifuged at 13,000 g at 4°C for 10 minutes. Globin chain separation was performed by injecting 10 μl of the supernatant onto a 1.0 × 250 mm C4 column (Phenomenex, Macclesfield, U.K., http://www.phenomenex.com/) with a 42%‐56% linear gradient between mixtures of 0.1% TFA in water (Buffer A) and 0.1% TFA in acetonitrile (Buffer B) at flow rate of 0.05 ml/min for 55 minutes on a HPLC Ultimate 3000 system (Dionex, Thermo Fisher Scientific Life Sciences). The column temperature was fixed at 50°C during analysis and the UV detector was set at 220 nm. Elution times of peaks generated were compared to control samples (e.g., adult and foetal blood) for identification and the area under the curve was used to calculate the proportion of each globin peak as a percentage of the total.

### Statistical Analysis

**Figure 1:**
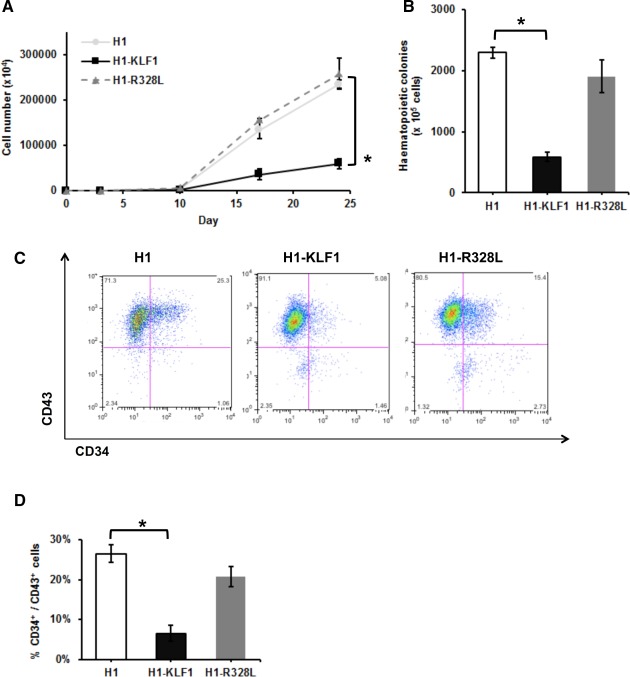
Constitutive KLF1 expression in human embryonic stem cells (hESCs) results in reduced proliferation and hematopoietic progenitor cell production. (**A)**: Cell counts throughout the erythroid differentiation protocol of control H1 hESCs (H1) and H1 hESCs transfected with a vector containing either wild type KLF1 (H1‐KLF1) or the mutant form of KLF1 (H1‐R328L). **(B)**: Total number of CFU‐Cs generated from differentiating H1, H1‐KLF1, and H1‐R328L hESCs at day 10 of the differentiation protocol. **(C)**: Flow cytometry analysis of differentiating H1, H1‐EKLF, and H1‐R328L hESC at day 10 of the differentiation protocol using antibodies against CD34 and CD43 to mark hematopoietic progenitor cells (HPCs). **(D)**: Quantification flow cytometry data showing the %CD34^+^/CD43^+^ HPCs at day 10 of the differentiation protocol. All data represents the mean of at least three independent experiments with error bars representing SEM. *p* values were calculated using two‐way ANOVA followed by Tukey's multiple comparisons test (A) or one‐way ANOVA followed by Holm‐Sidak's multiple comparison test (B and D) (*p < .05).

The statistical analysis was performed using GraphPad Prism 6 software. For cell proliferation (Figs. 1A, 4A) and globin expression by HPLC (Fig. 6), data were analyzed using two‐way ANOVA followed by Tukey's multiple comparisons test. CFU‐C (Fig. 1B) and flow cytometry data (Figs. 1D, 3D, 7C) were analyzed using one‐way ANOVA followed by Holm‐Sidak's multiple comparison test. Gene expression data were analyzed using ratio paired *t* test.

## Results

### Constitutive Overexpression of KLF1 in Differentiating hESCs Leads to Reduced Cell Proliferation and Hematopoietic Progenitor Cell Production

*KLF1* was expressed at a lower level in erythroid progenitors derived from hESC compared to those derived from adult peripheral blood CD34^+^ progenitors (Supporting Information Fig. S1A) and we hypothesized that this could be one of the reasons for their lack of maturity. H1 hESCs were transfected with vectors carrying either wild type KLF1 or mutant (R328L) KLF1 cDNA under the control of the constitutive CAG promoter followed by an intraribosomal entry site and the puromycin resistance gene (Supporting Information Fig. S1B). The R328L mutant protein had an arginine (R) to leucine (L) substitution in the second zinc finger domain at position 328 that abolishes activity in a transactivation assay (Supporting Information Fig. S1C) 29, but does not interfere with the activity of WT KLF1. There was no significant difference in the morphology of control H1, H1‐KLF1, and H1‐R328L hESC lines and all cell lines were maintained as undifferentiated hESCs in comparable conditions (data not shown). The morphology of transfected hESCs during the initial days of our erythroid differentiation protocol 15, 36 was comparable to parental H1 hESCs but the proliferation rate at later stages of the differentiation protocol was significantly lower in H1‐KLF1 cells (Fig. 1A). There was a significant reduction in the total number of CFU‐C colonies detected in H1‐KLF1 cells compared to control H1 cells and H1‐R328L cells (Fig. 1B). Flow cytometry confirmed the reduction in HPCs with fewer CD34^+^ CD43^+^ double positive cells generated in the H1‐KLF1 hESC line (Fig. 1C, 1D). Thus, constitutive expression of KLF1 resulted in a significant reduction in the proliferative capacity and an associated reduction in the production of HPCs hampering our ability to assess the specific effects of KLF1 on erythroid differentiation and maturation.

### KLF1‐ER^T2^ Fusion Protein Can Translocate to the Nucleus and Can Activate KLF1 Target Genes upon Induction

**Figure 2:**
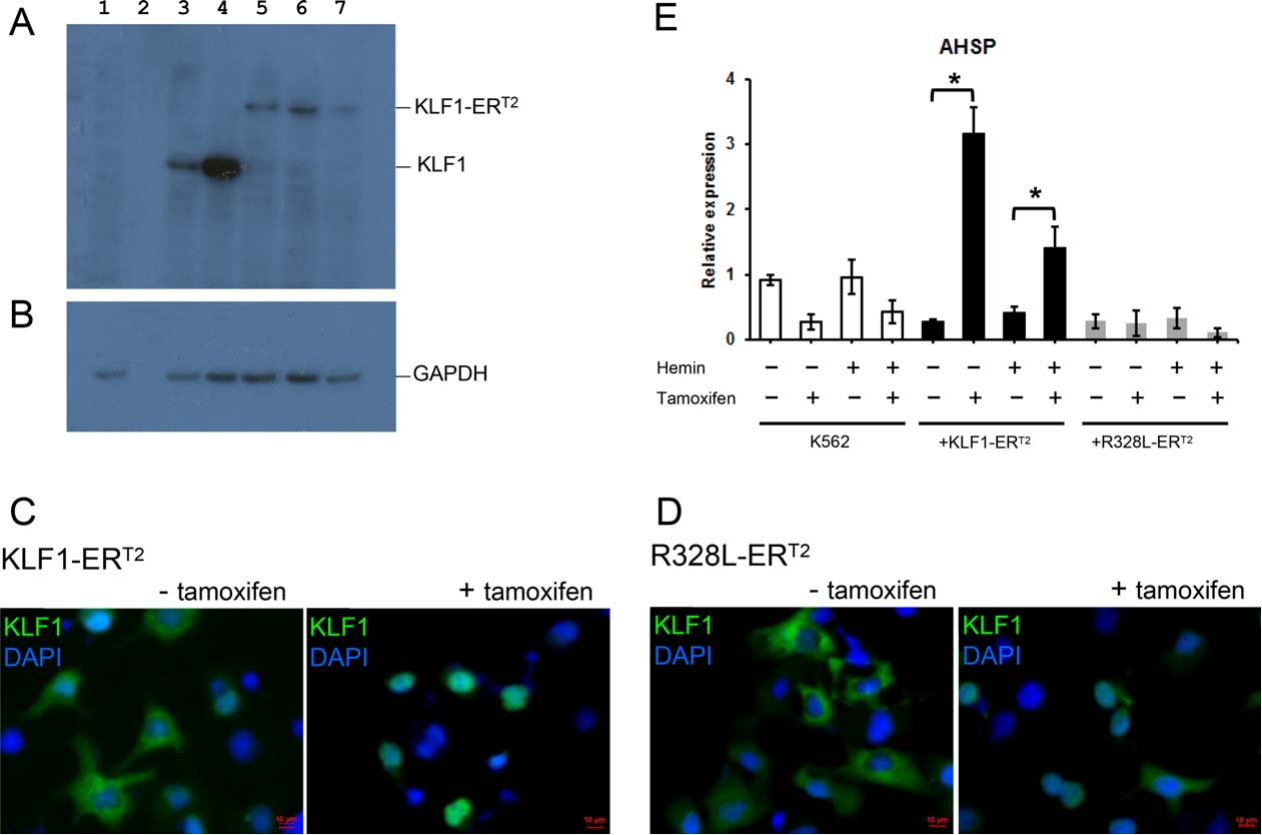
Functional assessment of KLF1‐ER^T2^ and R328L‐ER^T2^ fusion proteins. (**A, B)**: Western blot analyses of cell lysates isolated from untransfected COS7 cells (lane 1), COS7 cells transfected with pCAG‐KLF1 (lane 3); pCAG‐R328L (lane 4); pCAG‐KLF1‐ER^T2^ (lane 5); pCAG‐R328L‐ER^T2^ (lane 6) and the murine CAG‐ KLF1‐ER^T2^ (lane 7) using an anti‐KLF1 antibody (A) and GAPDH as a loading control (B). Lane 2 is blank. (**C, D)**: Immunofluorescence staining of COS7 cells transfected with either the CAG‐KLF1‐ER^T2^ (A) or CAG‐R328L‐ER^T2^ (B) constructs then stained with anti‐KLF1 antibodies (green) and the DAPI nuclear dye (blue) in the presence and absence of tamoxifen as indicated. (Scale bar 10 μm). **(E)**: Quantitative reverse‐transcriptase polymerase chain reaction analyses of RNA isolated from control and hemin and/or tamoxifen‐treated K562 cells and K562 cells transfected with either CAG‐KLF1‐ER^T2^ or CAG‐R328L‐ER^T2^ vectors using primers for the KLF1 target gene, *AHSP*. Data represent three independent experiments and error bars represent SEM. *p* values were calculated using using one‐way ANOVA followed Tukey's multiple comparisons test. (*p < .05).

We established an inducible strategy where we could activate KLF at specific time points during differentiation to assess the effects of this transcription factor on later erythroid cell production and maturation. We fused the human KLF1 and the mutant KLF1 (R328L) to the mutated form of the oestrogen receptor (ER^T2^) (Supporting Information Fig. S1D, S1E) and created the expected sized fusion protein of 74 kD (Fig. 2A, 2B). Before investing resources on assessing this strategy on hiPSCs, we first tested the functionality of the KLF1 inducible strategy on simpler well‐established cell systems. We used transiently transfected COS7 cells where high levels of transgene expression enable the subcellular location of fusion proteins to be assessed by immunofluorescence staining. This demonstrated that wild type KLF1‐ER^T2^ and mutant R328L‐ER^T2^ fusion proteins are sequestered in the cytoplasm and, upon tamoxifen treatment, they are released and can translocate to the nucleus (Fig. 2C, 2D).

To assess whether the KLF1‐ER^T2^ fusion protein could activate the expression of KLF1 target genes within a hematopoietic context, we used the K562 human leukemia cell line that could be induced to differentiate into the erythroid cells. Pools of puromycin‐resistant K562 cells were generated then RNA was isolated after culturing in the presence or absence of tamoxifen. Functionality of the KLF1‐ER^T2^ fusion protein was confirmed by demonstrating that the addition of tamoxifen enhanced the expression level of a known KLF1 target gene, Alpha Hemoglobin Stabilizing Protein (*AHSP*) (Fig. 2E). No significant increase in *AHSP* expression was observed after tamoxifen treatment of cells transfected with the CAG‐R328L‐ER^T2^ construct confirming the lack of transcriptional activity of the mutant form that had been predicted previously from luciferase assays (Supporting Information Fig. S1C). Comparable levels of KLF1‐ER^T2^ and CAG‐R328L‐ER^T2^ protein were produced, excluding the possibility that that the lack of activity of the mutant R328L‐ER^T2^ was due to a lower level of expression (Fig. 2A).

### Activation of KLF1 Promoted Erythroid Differentiation of hESC and iPSCs

**Figure 3:**
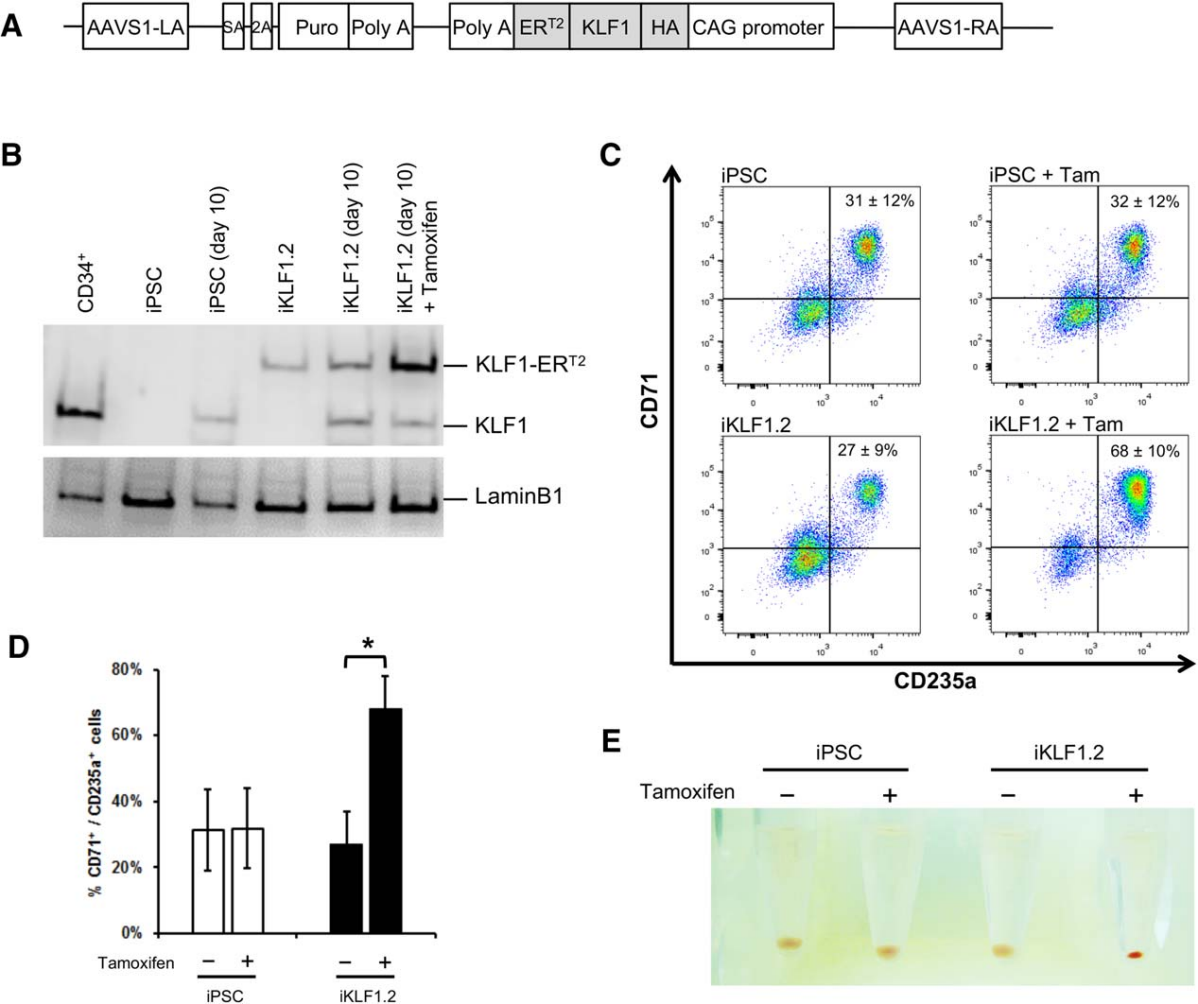
Activation of KLF1 at day 10 of differentiation results in enhanced erythroid differentiation of hiPSCs. **(A)**: Schematic of the pZDonor‐AAVS1 Puro‐CAG‐HA‐KLF1‐ER^T2^‐PA construct. **(B)**: Western blot analyses of nuclear cell lysates from adult CD34^+^ cells that had been differentiated for 6 days into erythroid progenitors, control undifferentiated and differentiated (day 10) induced pluripotent stem cells (iPSCs), undifferentiated iKLF1.2 iPSCs and iKLF1.2 iPSC that had been differentiated for 10 days then treated with tamoxifen for 3 hours. Endogenous KLF1 and the expected larger sized KLF1‐ER^T2^ fusion protein was detected with the anti‐KLF1 antibody and the anti‐Lamin B1 antibody was used to detect nuclear proteins as a loading control. **(C)**: Flow cytometry analysis using antibodies against CD235a and CD71 of cells present at day 15 of the erythroid differentiation protocol in control iPSCs and iKLF1.2 iPSC cell lines in the presence (+) and absence (−) of tamoxifen from day 10. (**D)**: Quantitation of flow cytometry data representing three independent experiments. Error bars represent SEM. *p* values were calculated using one‐way ANOVA followed by Holm‐Sidak's multiple comparison test (*p < .05). (**E)**: Image showing the cell pellets from one representative experiment demonstrating a smaller but more intense red pellet in the tamoxifen‐treated iKLF1.2 cell line. Abbreviation: iPSCs, induced pluripotent stem cells.

We then tested the effects of KLF1 activation on the production and maturation of erythroid cells during hESC and iPSC differentiation. Pilot experiments where pCAG‐KLF1‐ER^T2^ and pCAG‐R382R‐ER^T2^ constructs were randomly integrated into the genome of the H1 hESCs indicated that activation of KLF1 promoted the differentiation of erythroid cells as assessed by an increase in the proportion of CD235a^+^ CD71^+^ expressing cells and an increase in the level of CD235a expression (Supporting Information Fig. S3). However, given the known silencing issues associated with random integration of transgenes and the potential detrimental effects of insertion mutagenesis, we adopted a ‘safe harbor’ approach and targeted the CAG‐HA‐KLF1‐ER^T2^ transgene to the *AAVS1* locus (Fig. 3A) 34. We generated the AAVS1‐HA‐KLF1‐ER^T2^ targeting vector (Fig. 3A) and electroporated this together with the AAVS1 zinc finger nuclease (ZFN) plasmids (a gift from Dr C.J. Chang, Icahn School of Medicine at Mount Sinai, New York) 33, 35 into the human iPSC line, SFCi55 (Supporting Information Fig. S2). Puromycin‐resistant colonies were screened by genomic PCR (Supporting Information Fig. S4). 93% (27/29) of iPSC clones were correctly targeted with both *AAVS1* alleles targeted in 13 clones (Supporting Information Fig. S4A‐4G). Western blot analyses using the α−HA antibody detected the fusion protein in targeted iPSC clones (herein referred to as iKLF1.1 and iKLF1.2) (Supporting Information Fig. S5A). We confirmed the presence of the predicted sized KLF1‐ER^T2^ fusion protein in nuclear extracts isolated from undifferentiated and differentiated (day 10) iKLF1.2 iPSCs and noted that the level of expression of KLF1 protein in day 10 differentiating iPSCs was significantly lower than in adult CD34^+^ cells (Fig. 3B) as was the level of *KLF1* transcript (Supporting Information Fig. S5C) as previously demonstrated (Supporting Information Fig. S1). We noted the presence of a low level of KLF1‐ER^T2^ fusion protein in our crude nuclear extracts in the absence of tamoxifen but it is unclear whether this is due to cytoplasmic contamination or leakiness of the ER^T2^ system (Fig. 3B). Addition of tamoxifen for 3 hours resulted in the translocation of KLF1‐ER^T2^ protein into the nucleus (Fig. 3B). The level KLF1 protein expression in differentiating iKLF1.2 iPSCs is comparable to the level of expression of endogenous KLF1 in differentiating adult CD34^+^ cells indicating that, unlike lentiviral expression strategies that result in very high, nonphysiological levels of transgene expression, this strategy results in physiological levels of KLF1 (Fig. 3B, Supporting Information Fig. S5C).

**Figure 4:**
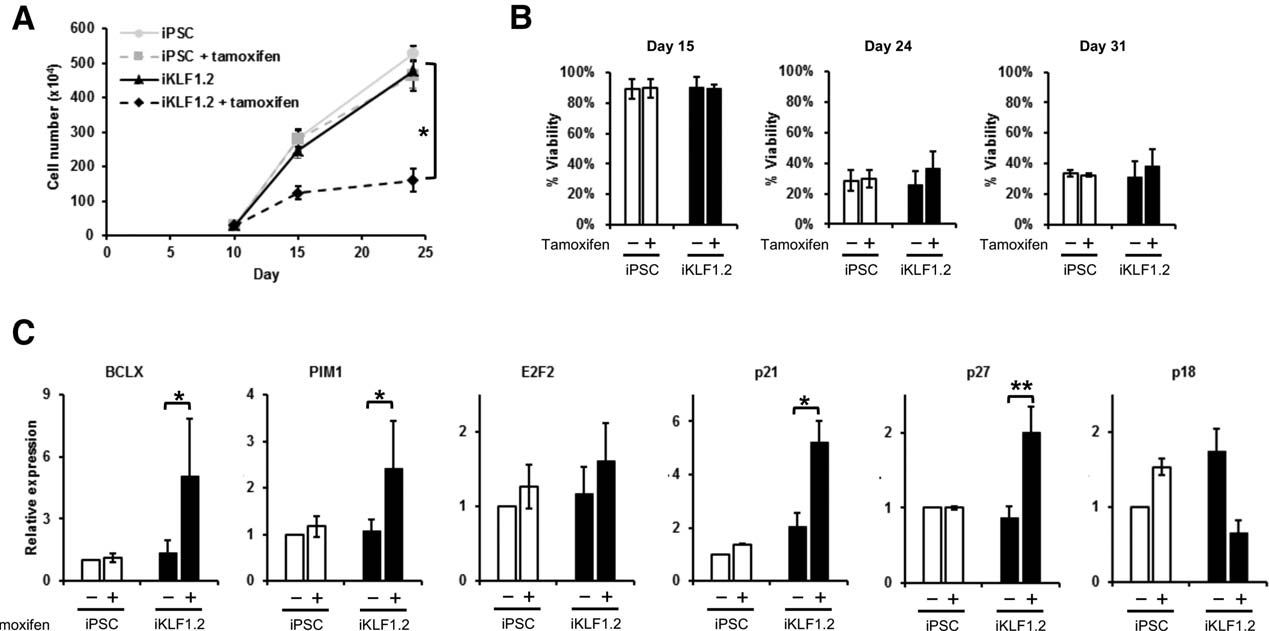
Activation of KLF1 enhances erythroid differentiation at the expense of cell proliferation. (**A)**: Cell numbers of control and iKLF1.2 induced pluripotent stem cells (iPSCs) during erythroid differentiation. 3 × 10^5^ cells were seeded at day 10 of differentiation then further differentiated in the presence or absence of tamoxifen. Data represent the mean of three independent experiments and error bars represents SEM. *p* values were calculated using two‐way ANOVA followed by Tukey's multiple comparisons test (*p < .05). (**B)**: Flow cytometry analysis using LIVE/DEAD Fixable Near‐IR Stain of viable cells present at day 15, 24, and 31 of the erythroid differentiation protocol in control iPSCs and iKLF1.2 iPSC cell lines in the presence (+) and absence (−) of tamoxifen from day 10. (**C)**: Quantitative reverse‐transcriptase polymerase chain reaction analyses of RNA isolated from control (iPSC) and iKLF1 iPSC (iKLF1.2) at day 24 following treatment with (+) or without (−) tamoxifen from day 10 using primers to *BCLX, PIM1* and *E2F2, p21, p27,* and *p18*. Data represent the mean of three independent experiments and error bars show the SEM. For each gene, the expression level of control iPSCs in the absence of tamoxifen was used as the calibrator and set at 1 and the expression of all other samples expressed as fold change. A ratio paired *t* test was used to assess the effect of KLF1 activation in iKLF1.2 cells (*p < .05 **p < .005). Abbreviation: iPSCs, induced pluripotent stem cells.

The clonal iKLF1.2 iPSC line was differentiated using the erythroid differentiation protocol 36 and we assessed the production of erythroid cells at day 15 in the presence and absence of tamoxifen (from day 10). Upon activation of KLF1, the percentage of CD235a^+^ CD71^+^ double positive erythroid cells increased in the iKLF1.2 iPSC lines, but not in control iPSCs (Fig. 3C, 3D). This increase was also observed in the independently derived iKLF1.1 cell line (Supporting Information Fig. S5B) and was consistent with the results using randomly inserted constructs in hESCs (Supporting Information Fig. S3A). These data indicate that activation of KLF1 enhanced erythroid differentiation, visually evident by the enhanced red appearance of the cell pellet (Fig. 3E). We also noted that tamoxifen treated cultures generated a smaller cell pellet and a significantly lower number of cells (Fig. 4A). There was no significant difference in cell viability at days 15, 24, and 31 when KLF1 was activated at day 10 indicating that the reduced cell number was not the result of increased apoptosis or cell death. (Fig. 4B). Furthermore, quantitative reverse‐transcriptase polymerase chain reaction analyses of differentiating cells (day 24) demonstrated that activation of KLF1 resulted in a significant upregulation of the cell cycle inhibitors *P21* and *P27*, the anti‐apoptotic gene, *BCLX* and *PIM1* that regulates cell proliferation and survival (Fig. 4C). Interestingly KLF1 activation did not result in the upregulation of *P18* which has been shown to mediate the effects of KLF1 effect on cell cycle exit in the murine system 39. Taken together, our data suggest that activation of KLF1 promotes erythroid differentiation at the expense of cell proliferation.

### Activation of KLF1 Enhanced the Expression of Genes Associated with Erythropoiesis

**Figure 5:**
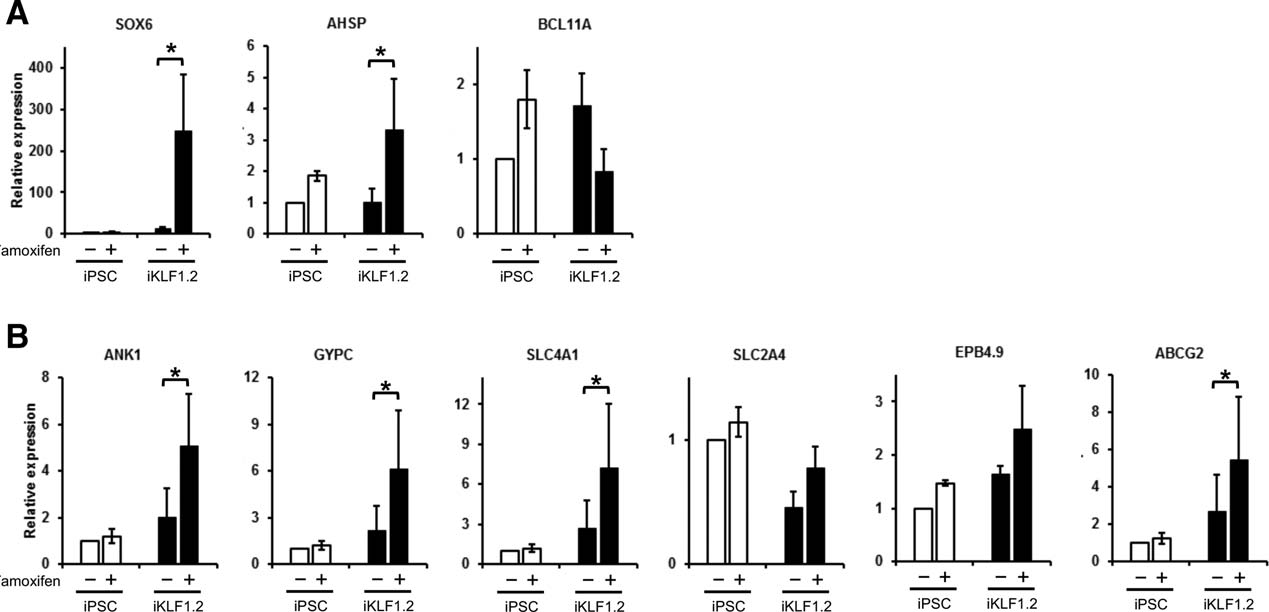
A subset of KLF1 target genes are upregulated upon activation of KLF1 during erythroid differentiation. Quantitative reverse‐transcriptase polymerase chain reaction analyses of RNA isolated from control induced pluripotent stem cell (iPSC) and iKLF1 iPSC (iKLF1.2) at day 15 (A) and day 24 (B) following treatment with (+) or without (−) tamoxifen from day 10 using primers to *SOX6, AHSP, BCL11A, ANK1, GYPC, SLC4A1, SLC2A4, EPB4.9,* and *ABCG2*. Data represent the mean of three independent experiments and error bars show the SEM. For each gene, the expression level of control iPSCs in the absence of tamoxifen was used as the calibrator and set at 1 and the expression of all other samples expressed as fold change. A ratio paired *t* test was used to assess the effect of KLF1 activation in iKLF1.2 cells (*p < .05). Abbreviation: iPSCs, induced pluripotent stem cells.

To investigate the impact of KLF1 activation on genes associated with erythropoiesis, we conducted real‐time PCR on RNA isolated from differentiating control hiPSCs and iKLF1.2 at day 15 in the presence or absence of tamoxifen from day 10 (Fig. 5A, Supporting Information Fig. S6A). Activation of KLF1 significantly increased the expression of the erythroid transcription factor‐encoding gene, *SOX6* consistent with the higher proportion of erythroid cells. Consistent with our findings in K562 cells, expression of the known KLF1 target gene *AHSP* was significantly upregulated upon KLF1 activation. Interestingly the expression of *BCL11A,* a known target gene of KLF1 was not enhanced in this assay but this is possibly explained by the fact that these cells have a “primitive”‐like signature (see below). The expression of KLF1 target genes associated with RBC maturation was also analyzed at a later stage in the differentiation process (day 24) (Fig. 5B, Supporting Information Fig. S6B). At this time point, expression of genes associated with cell membrane and cytoskeleton, including *ANK1*, *GYPC*, and *SLC4A1* were significantly increased upon KLF1 activation but there was no significant increase in the expression of *SLC2A4* nor *EPB4.9*. The expression of *ABCG2* that is involved in transport and heme synthesis was also upregulated by KLF1 activation. The above results suggest KLF1 activation from day 10 increased the maturity of erythroid cells and accelerated the process of erythropoiesis.

### KLF1‐Activated Erythroid Cells Express Embryonic Globins

**Figure 6:**
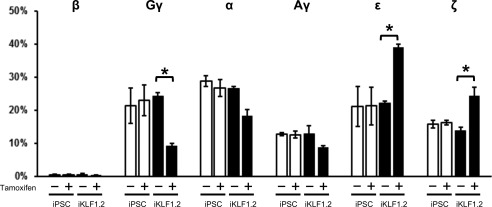
Activation of KLF1 enhances the production erythroid cells expressing embryonic globins. Results of HPLC analysis of globin proteins in cell lysates isolated from control induced pluripotent stem cells and iKLF1.2 cells at day 31 of the differentiation protocol in the presence (+) or absence (−) of tamoxifen from day 10. Data represent the mean of three independent experiments and error bars represents SEM. *p* values were calculated using two‐way ANOVA followed by Tukey's multiple comparisons test. (*p < .05). Abbreviation: iPSCs, induced pluripotent stem cells.

HPLC analyses of protein isolated from cells at day 31 of the differentiation protocol showed that activation of KLF1 in iKLF1.2‐derived erythroid cells significantly enhanced the proportion of the embryonic ε‐ and ζ‐globin and reduced the proportion of γ‐globin protein. No adult β‐globin protein was detected in any of the samples (Fig. 6). Taken together these data suggests that, in this differentiation system, activation of KLF1 at day 10 of the differentiation protocol enhances the production and maturation of primitive erythroid cells.

### KLF1 Activation Increases the Proportion of Enucleated Erythroid Cells

**Figure 7:**
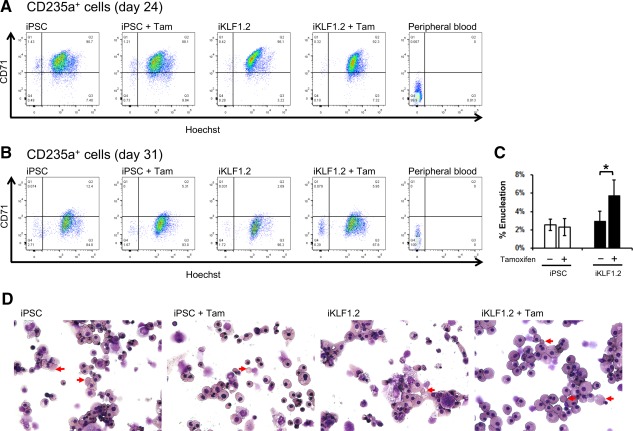
KLF1 activation increases the proportion of detectable enucleated cells. (**A, B)**: CD71 and Hoechst staining of live, CD235a gated cells at day 24 (A) and day 31 (B) derived from control induced pluripotent stem cell (iPSC) and KLF1‐ER^T2^‐expressing (iKLF1.2) cells in the presence and absence of tamoxifen. Control enucleated cells derived from adult peripheral blood is shown. (see Supporting Information Fig. S7 for gating strategy) **(C)** Quantification of the proportion of enucleated cells at day 31 of control iPSCs and iKLF1.2 cells in the presence and absence of tamoxifen. Data represent the mean of three independent experiments and error bars represents SEM. *p* values were calculated using one‐way ANOVA followed by Holm‐Sidak's multiple comparison test (*p < .05). (**D)**: Cytospins of day 31 cells demonstrating the more robust phenotype of iKLF1 cells after tamoxifen treatment and the presence of some enucleated cells (arrows) (Magnification x40). Abbreviation: iPSCs, induced pluripotent stem cells.

Given previous reports that KLF1 null mice had more nucleated RBCs 25, we hypothesized that activation of exogenous KLF1 might enhance the efficiency of maturation and/or their stability. Differentiating KLF1‐ER^T2^‐expressing cells were treated with tamoxifen then the presence of enucleate cells assessed by flow cytometry. Live CD235a^+^ cells were gated and CD235a^+^/CD71^+^/Hoechst^+^ erythroblasts, CD235a^+^/CD71^−^/Hoechst^+^ nucleated RBCs and CD235a^+^/CD71^−^/Hoechst^−^ enucleated RBCs were identified (Supporting Information Fig. S7). Human peripheral blood was used as a positive control for the identification of live, CD235a^+^/CD71^−^/Hoechst^‐^ enucleated RBCs. The majority of differentiating cells at day 24 were nucleated CD235a^+^/CD71^+^/Hoechst^+^ erythroblasts (Fig. 7A). By day 31 CD235a^+^ cells began to lose the CD71 marker, indicating that they represented a more mature erythroid population (Fig. 7B). Activation of KLF1 in differentiating iPSCs reproducibly increased the proportion of enucleated RBCs that were detected in this assay (Fig. 7B, 7C). Morphological analyses indicated that that KLF1‐activated cells had a more robust morphology which could explain the fact that more enucleated cells were detected (Fig. 7D).

## Discussion

Current protocols to produce RBCs from hPSC have limitations because they generate a relatively low proportion of enucleated cells and express embryonic/foetal but not adult globin 6. Forward programming using lineage specific transcription factors has been used to enhance the production of a number cell types from hPSCs including hematopoietic lineages 16, 17, 40. Here we describe the first application of an inducible programming strategy to modify the production and maturation of RBCs from hPSCs. We used the transcription factor KLF1 because it was expressed at low levels in hPSC‐derived erythroid cells compared adult‐derived cells and it plays a pivotal role in the final steps of definitive erythropoiesis 19. Genes that are regulated by KLF1 include many of the key genes associated with erythroid development and maturation 19, 25, 41, 42.

We established an inducible activation strategy whereby the exogenous KLF‐ER^T2^ fusion protein is tethered in the cytoplasm but upon addition of tamoxifen it can translocate to the nucleus and activate the expression of target genes. The level expression of KLF1 in differentiating iKLF1.2 iPSCs is comparable to the level of expression in differentiating adult CD34^+^ cells indicating that physiological levels of expression are achieved using this strategy.

Activation of KLF1 at day 10 after HPC formation resulted in an increase in the proportion of erythroid cells but the overall number of cells was lower than controls. The fact that we observed no effect on the viability of cells suggests that activation of KLF1 is driving HPCs to differentiate at the expense of proliferation 43. It is well documented that erythroid terminal differentiation requires proliferation arrest and exit from the cell cycle with a balance between proliferation and maturation being fine‐tuned at later stages of erythropoiesis 24, 44. The antiproliferative effect of KLF1 during erythropoiesis is thought to be via its interactions with cell cycle related genes including *PIM1, E2F2, p27*, *p21, p18* 19, 39, 45. We demonstrate that activation of KLF1 significantly altered the expression levels of *p27*, *p21,* and *PIM1* but not *p18* suggesting that some, but not all, of these interactions are conserved between mouse and human. Our results are consistent with a study using a similar KLF1‐ER^T2^ strategy in murine ESCs where activation of KLF1 resulted in reduced proliferation coupled with enhanced differentiation 46. Another study using a tetracycline‐inducible KLF1 strategy in murine ESCs expression reported that KLF1 promoted the expression of erythroid lineage genes while repressing the onset of megakaryopoiesis 43. We detected an increase in expression of KLF1 target genes associated with heme synthesis and transport including *ABCG2* and *AHSP*, supporting the notion that KLF1‐activation enhanced the erythroid maturation and differentiation process.

Activation of exogenous KLF1 resulted in an increase in the proportion of detectable enucleated erythroid cells. It has been proposed that hPSC‐derived erythroid cells may be more fragile than their counterparts generated from adult CD34 progenitors 36 and so it is possible that the effect of KLF1 is due to enhanced membrane stability rather than a direct effect on the enucleation process per se. The final stages of RBC maturation are associated with cell membrane and cytoskeleton remodelling and a number of KLF1 target genes have been associated with these processes 19, 21, 26, 47. Furthermore, the phenotype of KLF1 deficient mice has been associated with decreased membrane stability 21. Activation of KLF1 in our system enhanced the expression of some of these KLF1 targets including *ANK1, GYPC, SLC4A1*, and *ABCG2* which supports our hypothesis that activation of KLF1 results in the production of more robust erythroid cells.

The mechanisms of enucleation is known to involve multiple molecular and cellular pathways including histone deacetylation, actin polymerization, cytokinesis, cell‐matrix interactions, specific microRNAs, and vesicle trafficking 48. Enucleation efficiency of iPSC‐derived erythroid cells was improved with stromal cell culture and when cells were derived from cultures involving prolonged three‐dimensional culture 1, 5. More recently KLF1 has been shown to have an extrinsic role in erythroid maturation via expression of KLF1 in erythroid‐island associated macrophages 49, 50 so KLF1 may be playing an extrinsic role during the differentiation process. It is also possible that KLF1 activation is altering the expression of miRNAs or long noncoding RNAs that have been identified as key players in erythroid development and maturation 1, 51.

The majority of hPSC differentiation protocols generate RBCs that express embryonic ε‐globins and/or foetal γ‐globins, but little or no adult β‐globin 6, 7. We show that KLF1 enhanced the expression of embryonic ε‐ and ζ‐globin proteins, but no adult β‐globin was detected in any of the conditions suggesting that this strategy is enhancing the production of embryonic erythroid cells and that KLF1 alone is not sufficient to enhance the expression of adult β‐globin. A low level of expression of KLF1 and BCL11A in K562 cell and cord blood derived erythroid cells was shown to be associated with fetal globlin expression and transduction of *KLF1* and *BCL11A* lentiviral vectors resulted in adult levels of β−globin in these cells 52. Interestingly that study also demonstrated that lentiviral transduction of *BCL11A* alone was sufficient to induce the expression of β−globin in the immortalized iPSC‐derived HiDEP‐1 cell line because that cell lines had adult‐like levels of KLF1 52. A recent study that added KLF1 to iEP cells showed adult‐like globin 17. The erythroid cells derived from the SFCi55 iPSC cell line used in this study have lower levels of KLF1 compared to differentiated adult CD34^+^ cells (Fig. 3B, Supporting Information Fig. S5C) and, although *BCL11A* has been reported as a KLF1 target gene, we did not see a significant alteration in the level of expression of *BCL11A* upon KLF1 activation in our system. Activation of KLF1 target genes will rely on the presence of specific cofactors which will be cell context dependent. More recent studies highlight the complexity of interaction between KLF1 and its regulated genes and specialized transcription factories in nuclear hotspots have been identified that are likely where coregulated genes cooperate for optimal efficiency and coordinated transcriptional control 20. Our ongoing studies are assessing the effects of exogenous expression of both *KLF1* and *BCL11A* on the expression of the different globin proteins in differentiating iPSCs.

Flow cytometry analyses of erythroid markers throughout the differentiation protocol indicates that there are two waves of erythropoiesis in our culture system 36 and our data suggest that activation of KLF1 at day 10 is enhancing the primitive rather than the definitive wave.

This study is the first to demonstrate enhanced erythropoiesis from hPSCs using a forward programming approach by activation of a single transcription factor, KLF1 at levels that are comparable to physiological level. However, the successful production of adult‐like erythroid cells in sufficient quantities from iPSCs will undoubtedly require the use of multiple transcription factors in a combinatorial forward programming approach as recently described for the production of platelets form hPSCs 40 and primitive erythroid cells from fibroblasts 17. The key to this strategy is to define the complex cocktail of transcription factors that define the development and maintenance of adult erythroid cells and to induce their expression at defined time‐points in a reproducible manner. Integration of inducible transcription factors into the *AAVS1* locus could provide a safer and more reproducible strategy for clinical translation.

This study assessed the effects of KLF1 on the production and maturation of erythroid cells from differentiating human pluripotent stem cells. We generated a human iPSC line carrying a tamoxifen‐inducible form of KLF1 in the AAVS1 locus. Activation of KLF1 alone promoted erythroid differentiation, enhanced the expression of key erythroid genes and generated a slightly higher proportion of mature enucleated cells. However, activation of KLF1 promoted the differentiation of primitive, not definitive erythroid cells as defined by an increase in embryonic globins. The enhanced erythroid differentiation is associated with a proliferation arrest and the upregulation of the cell cycle inhibitors P21 and P27 resulting in a significant reduction in the overall number of cells generated.

## Author Contributions

C.‐T.Y.: Performed research, analysed data and wrote paper; R.M.: Performed research and analysed data; R.A.A.: Performed research; M.J.: performed research; A.H.T.: Performed research; A.F.: Performed research and analysed data; L.M.: Performed research; J.F., J.C.M.: Designed research and analysed data; L.M.F.: Designed research, analysed data and wrote paper.

## Disclosure of Potential Conflicts of Interest

The authors indicate no potential conflicts of interest.

## Supporting information


**Supplementary Figure S1. A**. Relative expression of KLF1 extracted from microarray data of erythroid cells derived either from hESCs (RC9 and H1) and adult peripheral blood (adult). P values were calculated using one‐way ANOVA followed by Dunn's multiple comparison test. (*, p<0.05). Microarray data was confirmed by qRT‐PCR (data not shown).
**B**. Schematic of vectors used to express constitutive wild type KLF1 / mutant R328L cDNAs under the control of the CAG promoter linked to puromycin resistance gene by intra‐ribosomal entry site (IRES).
C. The R328L KLF1 mutant is nonfunctional in a β‐globin promoter‐*f*luc assay and does not interfere with activity of wild type KLF1.
Relative promoter activity in K562 cell extracts 24 hours after transient co‐transfection with β‐globin promoter‐*f*luc construct, pRL CMV and 5mg of either empty vector (EV) or constructs expressing wild type (WT) or R328L mutant EKLF (R328L). Relative promoter activity is expressed as the firefly luciferase activity normalised for transfection efficiency using renilla luciferase activity. Results are shown as means +/‐ SD (n=3).
**D**. Schematic of vectors used to express the inducible wild type KLF1‐ER^T2^ / mutant R328L‐ER^T2^ cassettes under the control of the CAG promoter linked to puromycin resistance gene by intra‐ribosomal entry site (IRES).
The ER^T2^ domain was first amplified using Primers 1 and 3 then this PCR product was mixed with KLF1 cDNA and primers 2 and 3 and the KLF1‐ERT2 was amplified using FailSafe PCR (Epicentre) with the premix J solution (FS99100) according to manufacturer's instructions.
*Primer 1: primer with an KLF1 sequence at the 5'end of the ER^*T2*^ sequence*.
Primer 2: primer with an EcoRI site at the 5'end of the KLF1 sequence.
*Primer 3: primer with an EcoRI site at the 3'end of the ER^*T2*^ sequence*.
Supplementary Figure S2. Characterisation of O RhesusD negative iPSCs.
**A/B**. Flow cytometry analyses of SFCi55 iPSC line of pluripotency markers (TRA1‐60, SSEA‐4, OCT4) and differentiation marker (SSEA‐1).
**C**. CFU‐C formation indicative of the hematopoietic differentiation potential of SFCi55 iPSCs was compared to a number of other iPSC lines was assessed at day 10 of the differentiation protocol (Olivier et al 2016).
**Supplementary Figure S3. Inducible KLF1‐ER^**T2**^ system in H1‐ESCs**.
A. Percentage of CD235a and CD71 double positive cells in the presence or absence of 200nM tamoxifen at day 17.
B. Mean Fluorescence intensity (MFI) measurements of CD235a expression in day 17 cells following administration of 0 (control) 100 or 200nM tamoxifen from day 10 of differentiation.
All data represent the mean of 3 independent experiments with error bars showing the standard error of the mean (SEM). P values were calculated using paired t‐test (*p<0.05)
Supplementary Figure S4. AAVS targeting strategy
A. Schematic of genomic structure of targeted alleles showing the locations of diagnostic internal and external PCR assays, 1‐3.
B‐D. Genomic PCR analyses using internal primer pair (PCR 1) demonstrating integration of the vector (B) and diagnostic PCR confirming correct targeting at the 5′ (PCR 2)(C) and 3′ end (PCR 3)(D) in 27/29 clones.
E. Schematic of genomic structure of the endogenous, untargeted AAVS locus.
F. Genomic PCR analysis using primer pair, PCR 4 distinguished homozygous and heterozygous targeted events. Note that the two clones (no 4 and 27) that were not targeted (identified in C and D above) generated a more intense PCR product in this untargeted PCR assay as predicted.
Thus 13/29 of the clones were targeted at both AAVS alleles (homozygous), 14/29 at one allele (heterozygous) and 2/29 were not targeted.
G. PCR genotypes of 2 homozygous (iKLF1.1 and iKLF 1.2) and 2 heterozygous (iKLF1.19 and iKLF 1.25) were confirmed using the 4 PCR assays, 1‐4.
AAVS1‐RA, AAVS1 right homology arm; SA, splice acceptor; 2A, a self‐cleaving peptide sequence; Puro, puromycin resistance gene; PolyA, polyadenylation sequence; AAVS1‐LA, AAVS1 left homology arm.
Supplementary Figure S5. KLF1 expression and production of erythroid cells from control iPSCs and iPSC lines iKLF1.1 and iKLF1.2.
A. Western blot analyses of cell lysates from control iPSCs (Con) and two puromycin‐resistant iKLF1 iPSC clones (iKLF1.1 and iKLF1.2) using anti‐HA (αHA), anti‐KLF1 (αKLF1) and anti‐GAPDH (αGAPDH) antibodies. (The band observed in Control sample with the αHA antibody is non‐specific)
B. %CD235a+/CD71+ cells in day 15 differentiated control iPSCS and in two independently‐derived iKLF1 cell lines (iKLF1.1 and iKLF1.2) in the presence (+) and absence (‐) of tamoxifen from day 10 to day 15. This experiment was performed once on iKLF1.1 and iKLF1.2 so no error bars are shown. Three repeat experiments on iKLF1.2 are shown in Figure 3D.
C, D. Expression of KLF1 and KLF1‐ER^T2^ in CD34+ cells, undifferentiated iPSCs and iKLF1.2 cells and iPSCs and iKLF1.2 cells differentiated for 10 days in the erythroid differentiation protocol when the majority of CD34^+^ cells are present. Note the lower level of endogenous KLF1 in iPSCs compared to adult CD34^+^ cells and that the expression of KLF1 in transgenic iKLF1.2 cells is significantly higher than in control iPSCs at same stage in differentiation protocol. Real time PCR analyses was carried out with primers to KLF1 (C) that amplifies both endogenous and exogenous KLF1 and primers that amplify only the KLF1‐ER^T2^ exogenous transgene (D)
Supplementary Figure S6. Tamoxifen has no effect on the expression levels of KLF1 target genes in control iPSCs
In Figure 4, statistical analysis of the data to assess the effects of KLF1 activation in iKLF1 cells was performed by using the gene expression level of control iPSCs as the calibrator (set as 1) precluding a valid statistical analysis of these control cells. To demonstrate that tamoxifen had minimal effect of the expression level of these genes in control iPSCs we calculated the ‘fold change’ in these cells in the presence and absence of tamoxifen using iKLF1 (no tamoxifen) as the calibrator. A ratio paired T test was used to assess the effect of tamoxifen in iPSC derived cells (*, p<0.05).
Samples are as Figure 4 from day 15 (A) and Day 24 (B).
Supplementary Figure S7. Flow cytometry gating strategy for the enucleation assay
Differentiating cells were stained with anti‐CD235a, ‐CD71 antibodies, Hoechst and the LIVE/DEAD™ Fixable Near‐IR Stain then analysed by flow cytometry. Single cells were gated by FSC‐A and FSC‐H, and live cells were identified by the R780_60 filter. Gating thresholds were all set using the appropriate FMO and live CD235a positive cells were then assessed in the enucleation assay. Similarly, the gating thresholds for CD71 and Hoechst were set using the appropriate FMOs minus CD71 antibody and minus Hoechst. CD235a^+^ / CD71¯ / Hoechst¯ enucleated RBCs were expected to appear in Q4 quadrant. Control human peripheral blood was used as a positive control for enucleated RBCs.
