# Supplementary material for: Activation of KLF1 Enhances the Differentiation and Maturation of Red Blood Cells from Human Pluripotent Stem Cells
Source: Stem Cells. 2017 Jan 19;35(4):886–97. doi: 10.1002/stem.2562 (PMC5396323; doi:10.1002/stem.2562)
Supplement: Supplementary file 1 — Supplementary Figure S1. A. Relative expression of KLF1 extracted from microarray data of erythroid cells derived either from hESCs (RC9 and H1) and adult peripheral blood (adult). P values were calculated using one‐way ANOVA followed by Dunn's multiple comparison test. (*, p<0.05). Microarray data was confirmed by qRT‐PCR (data not shown). B. Schematic of vectors used to express constitutive wild type KLF1 / mutant R328L cDNAs under the control of the CAG promoter linked to puromycin resistance gene by intra‐ribosomal entry site (IRES). C. The R328L KLF1 mutant is nonfunctional in a β‐globin promoter‐fluc assay and does not interfere with activity of wild type KLF1. Relative promoter activity in K562 cell extracts 24 hours after transient co‐transfection with β‐globin promoter‐fluc construct, pRL CMV and 5mg of either empty vector (EV) or constructs expressing wild type (WT) or R328L mutant EKLF (R328L). Relative promoter activity is expressed as the firefly luciferase activity normalised for transfection efficiency using renilla luciferase activity. Results are shown as means +/‐ SD (n=3). D. Schematic of vectors used to express the inducible wild type KLF1‐ERT2 / mutant R328L‐ERT2 cassettes under the control of the CAG promoter linked to puromycin resistance gene by intra‐ribosomal entry site (IRES). The ERT2 domain was first amplified using Primers 1 and 3 then this PCR product was mixed with KLF1 cDNA and primers 2 and 3 and the KLF1‐ERT2 was amplified using FailSafe PCR (Epicentre) with the premix J solution (FS99100) according to manufacturer's instructions. Primer 1: primer with an KLF1 sequence at the 5'end of the ERT2 sequence. Primer 2: primer with an EcoRI site at the 5'end of the KLF1 sequence. Primer 3: primer with an EcoRI site at the 3'end of the ERT2 sequence. Supplementary Figure S2. Characterisation of O RhesusD negative iPSCs. A/B. Flow cytometry analyses of SFCi55 iPSC line of pluripotency markers (TRA1‐60, SSEA‐4, OCT4) and differentiation mark [file STEM-35-886-s001.docx]

**Yang et al. Supplementary Figures.**

**Supplementary Figure S1.**

**A**. Relative expression of KLF1 extracted from microarray data of erythroid cells derived either from hESCs (RC9 and H1) and adult peripheral blood (adult). P values were calculated using one-way ANOVA followed by Dunn’s multiple comparison test. (*, p<0.05). Microarray data was confirmed by qRT-PCR (data not shown).

**B**. Schematic of vectors used to express constitutive wild type KLF1 / mutant R328L cDNAs under the control of the CAG promoter linked to puromycin resistance gene by intra-ribosomal entry site (IRES).

**C. The R328L KLF1 mutant is nonfunctional in a β-globin promoter-*f*luc assay and does not interfere with activity of wild type KLF1.**

Relative promoter activity in K562 cell extracts 24 hours after transient co-transfection with β-globin promoter-*f*luc construct, pRL CMV and 5mg of either empty vector (EV) or constructs expressing wild type (WT) or R328L mutant EKLF (R328L). Relative promoter activity is expressed as the firefly luciferase activity normalised for transfection efficiency using renilla luciferase activity. Results are shown as means +/- SD (n=3).

**D**. Schematic of vectors used to express the inducible wild type KLF1-ER^T2^ / mutant R328L-ER^T2^ cassettes under the control of the CAG promoter linked to puromycin resistance gene by intra-ribosomal entry site (IRES).

The ER^T2^ domain was first amplified using Primers 1 and 3 then this PCR product was mixed with KLF1 cDNA and primers 2 and 3 and the KLF1-ERT2 was amplified using FailSafe PCR (Epicentre) with the premix J solution (FS99100) according to manufacturer’s instructions.

*Primer 1: primer with an KLF1 sequence at the 5’end of the ER^T2^ sequence.*

*Primer 2: primer with an EcoRI site at the 5’end of the KLF1 sequence.*

*Primer 3: primer with an EcoRI site at the 3’end of the ER^T2^ sequence.*

**Supplementary Figure S2. Characterisation of O RhesusD negative iPSCs.**

**A/B.** Flow cytometry analyses of SFCi55 iPSC line of pluripotency markers (TRA1-60, SSEA-4, OCT4) and differentiation marker (SSEA-1).

**C**. CFU-C formation indicative of the hematopoietic differentiation potential of SFCi55 iPSCs was compared to a number of other iPSC lines was assessed at day 10 of the differentiation protocol (Olivier et al 2016).

**Supplementary Figure S3. Inducible KLF1-ER^T2^ system in H1-ESCs.**

A**.** Percentage of CD235a and CD71 double positive cells in the presence or absence of 200nM tamoxifen at day 17.

B**.** Mean Fluorescence intensity (MFI) measurements of CD235a expression in day 17 cells following administration of 0 (control) 100 or 200nM tamoxifen from day 10 of differentiation.

All data represent the mean of 3 independent experiments with error bars showing the standard error of the mean (SEM). P values were calculated using paired t-test (*p<0.05)

**Supplementary Figure S4. AAVS targeting strategy**

A. Schematic of genomic structure of targeted alleles showing the locations of diagnostic internal and external PCR assays, 1-3.

B-D. Genomic PCR analyses using internal primer pair (PCR 1) demonstrating integration of the vector (B) and diagnostic PCR confirming correct targeting at the 5’ (PCR 2)(C) and 3’ end (PCR 3)(D) in 27/29 clones.

E. Schematic of genomic structure of the endogenous, untargeted AAVS locus.

F. Genomic PCR analysis using primer pair, PCR 4 distinguished homozygous and heterozygous targeted events. Note that the two clones (no 4 and 27) that were not targeted (identified in C and D above) generated a more intense PCR product in this untargeted PCR assay as predicted.

Thus 13/29 of the clones were targeted at both AAVS alleles (homozygous), 14/29 at one allele (heterozygous) and 2/29 were not targeted.

G. PCR genotypes of 2 homozygous (iKLF1.1 and iKLF 1.2) and 2 heterozygous (iKLF1.19 and iKLF 1.25) were confirmed using the 4 PCR assays, 1-4.

AAVS1-RA, AAVS1 right homology arm; SA, splice acceptor; 2A, a self-cleaving peptide sequence; Puro, puromycin resistance gene; PolyA, polyadenylation sequence; AAVS1-LA, AAVS1 left homology arm.

**Supplementary Figure S5. KLF1 expression and production of erythroid cells from control iPSCs and iPSC lines iKLF1.1 and iKLF1.2.**

A. Western blot analyses of cell lysates from control iPSCs (Con) and two puromycin-resistant iKLF1 iPSC clones (iKLF1.1 and iKLF1.2) using anti-HA (αHA), anti-KLF1 (αKLF1) and anti-GAPDH (αGAPDH) antibodies. (The band observed in Control sample with the αHA antibody is non-specific)

B. %CD235a+/CD71+ cells in day 15 differentiated control iPSCS and in two independently-derived iKLF1 cell lines (iKLF1.1 and iKLF1.2) in the presence (+) and absence (-) of tamoxifen from day 10 to day 15. This experiment was performed once on iKLF1.1 and iKLF1.2 so no error bars are shown. Three repeat experiments on iKLF1.2 are shown in Figure 3D.

C, D. Expression of KLF1 and KLF1-ER^T2^ in CD34+ cells, undifferentiated iPSCs and iKLF1.2 cells and iPSCs and iKLF1.2 cells differentiated for 10 days in the erythroid differentiation protocol when the majority of CD34^+^ cells are present. Note the lower level of endogenous KLF1 in iPSCs compared to adult CD34^+^ cells and that the expression of KLF1 in transgenic iKLF1.2 cells is significantly higher than in control iPSCs at same stage in differentiation protocol. Real time PCR analyses was carried out with primers to KLF1 (C) that amplifies both endogenous and exogenous KLF1 and primers that amplify only the KLF1-ER^T2^ exogenous transgene (D)

**Supplementary Figure S6. Tamoxifen has no effect on the expression levels of KLF1 target genes in control iPSCs**

In Figure 4, statistical analysis of the data to assess the effects of KLF1 activation in iKLF1 cells was performed by using the gene expression level of control iPSCs as the calibrator (set as 1) precluding a valid statistical analysis of these control cells. To demonstrate that tamoxifen had minimal effect of the expression level of these genes in control iPSCs we calculated the ‘fold change’ in these cells in the presence and absence of tamoxifen using iKLF1 (no tamoxifen) as the calibrator. A ratio paired T test was used to assess the effect of tamoxifen in iPSC derived cells (*, p<0.05).

Samples are as Figure 4 from day 15 (A) and Day 24 (B).

**Supplementary Figure S7. Flow cytometry gating strategy for the enucleation assay**

Differentiating cells were stained with anti-CD235a, -CD71 antibodies, Hoechst and the LIVE/DEAD™ Fixable Near-IR Stain then analysed by flow cytometry. Single cells were gated by FSC-A and FSC-H, and live cells were identified by the R780_60 filter. Gating thresholds were all set using the appropriate FMO and live CD235a positive cells were then assessed in the enucleation assay. Similarly, the gating thresholds for CD71 and Hoechst were set using the appropriate FMOs minus CD71 antibody and minus Hoechst. CD235a^+^ / CD71¯ / Hoechst¯ enucleated RBCs were expected to appear in Q4 quadrant. Control human peripheral blood was used as a positive control for enucleated RBCs.
